# mtDNA Mutation Accumulation in Muscle Is Not a Major Cause of Fiber Loss. Reply to “Comment on: Mitochondrial Mechanisms of Neuromuscular Junction Degeneration with Aging. *Cells* 2020, *9*, 197”

**DOI:** 10.3390/cells9081821

**Published:** 2020-08-01

**Authors:** Russell T. Hepple

**Affiliations:** Department of Physical Therapy, University of Florida, Gainesville, FL 32610, USA; rthepple@ufl.edu

I thank you for your comment on our review paper [1]. To be clear, I am not dismissing a role for mtDNA mutation accumulation in aging altogether, but rather specifically challenge the importance of mtDNA mutation accumulation within muscle fibers as a *quantitatively* important cause of muscle atrophy. Furthermore, I do not contest the observations of Lexell and colleagues showing that a loss of muscle fibers in aging muscle is an important element of the atrophy of aging muscle [2]; I am inspired by this data, too. I point out that our focus on muscle fiber atrophy is based upon the hypothesis that mtDNA deletions cause muscle fiber death by manifesting first as segmental atrophy at fiber regions with high mtDNA mutation burden [3]. Thus, for mtDNA mutations to be a significant cause of fiber death with aging, they should have a consistent association with fiber atrophy, too. It is established that prolonged muscle denervation leads to progressive fiber atrophy and eventual loss of muscle fibers [4], so there are other examples linking fiber atrophy and death as part of the same continuum. We did not intend for this focus on atrophy to be interpreted as a conflation of atrophy and fiber loss and should have explained that assumption.

The work from Aiken and colleagues set a high bar for technical interrogation of how mtDNA mutations may contribute to aging-muscle atrophy [3,5,6,7,8]. I have tremendous respect for the rigor of the methods employed and the quality of the data produced. Where I differ is in the interpretation of that data in the context of explaining aging-muscle atrophy. I accept the links drawn between mtDNA mutation load and resulting mitochondrial defects [3,7] and the activation of cell death and necrotic pathways [8]. However, based upon published data from Aiken’s group, only 5% of fibers with Cox deficiency exhibit intrafiber atrophy at the affected segment [6], suggesting that under most circumstances the Cox deficient muscle fiber/segment is able to compensate without inducing apoptotic or necrotic pathways. This leaves the question: are there enough fibers that cannot compensate for high mtDNA mutation load over the course of a lifetime to explain the atrophy seen in advanced age? Based on my reading of the literature, this does not seem likely because muscle fibers with high mtDNA mutation loads do not appear to be sufficiently vulnerable to atrophy or death to explain the muscle loss with aging.

To address your point about muscle atrophy in mtDNA disease, whilst mtDNA disease patients can have smaller muscle fibers (Table 3 in [9] indicates mean fiber cross-sectional area is 20% smaller in mtDNA disease patients than age-matched controls), Cox negative fibers and Cox normal fibers within a given cross-section visually have similar size (Figure 3 in [9]), showing that the atrophy in these patients generally affects all fibers rather than being specific to those with high mtDNA mutation load. This does not rule out occasional atrophy and death of affected fibers—as Dr. Aiken’s work has observed with normal aging [3,6,8]—but underscores the point that a high mtDNA mutation load within a muscle fiber is not a consistent recipe for its imminent death. Similar observations of a lack of atrophy in Cox deficient fibers have been reported by several labs examining aging muscle (e.g., [10,11]), in addition to ours [12] and Dr. Aiken’s [6]. Indeed, Cox deficient fibers not only appear quite atrophy-resistant, but a case-history study showed that Cox deficient fibers exhibited nearly 50% *hypertrophy* in response to resistance training exercise in a patient with mitochondrial disease [13]. It is hard to imagine that a muscle fiber that exhibits such impressive hypertrophic capacity is on the brink of death due to its high mtDNA mutation load. This speaks to the adaptive reserve still present in the majority of Cox deficient fibers. 

Based upon the literature, a 60-year-old human has approximately 1–2% of Cox deficient muscle fibers within a given cross-section, whereas it is not uncommon for a mtDNA disease patient 20 years younger to have 50–90% Cox deficient fibers in a cross-section [9]. Following the reasoning articulated by our colleagues [14], the number of Cox deficient fibers within a given muscle cross-section is only a fraction of all the affected fibers, suggesting all muscle fibers in mtDNA disease patients are likely to be affected at some point along their length. Thus, if mtDNA mutations were causal of fiber death and atrophy in a way that could quantitatively explain the 40% loss of muscle in a cross-sectional area between the age of 20 and 83 yr with normal aging [2], would we not expect much more rapid and severe atrophy in the mtDNA-disease patient given their conservatively 25-fold greater burden of Cox deficient fibers compared to normal aging? To my knowledge, this has not been demonstrated. Indeed, the massive accumulation of fibers with high mtDNA mutation loads in mtDNA disease also argues against these fibers being particularly susceptible to cell death. 

Finally, previous studies have shown that extraocular muscle is one of a very small number of skeletal muscles that are relatively resistant to atrophy and deterioration with aging in rodents [15,16] and humans [17], yet extraocular muscle has much higher levels of Cox deficient muscle fibers (which were demonstrated to harbor high mtDNA mutation loads) than limb skeletal muscle in the same aged individuals [18]. If this higher burden of Cox deficient fibers is benign to the extraocular muscle, why is limb skeletal muscle within the same individual vulnerable to atrophy at a lower Cox deficiency burden? Interestingly, however, extraocular muscle is often profoundly affected in patients with mtDNA disease where mutation loads at the whole muscle level are orders of magnitude greater than with aging [19]. While this shows mtDNA mutations can become a quantitatively meaningful cause of muscle deterioration when a large abundance of muscle fibers are severely affected, the much lower levels of mtDNA mutation/Cox deficiency associated with normal aging in extraocular muscle [18] appear to be well-tolerated. 

I acknowledge that there may be gaps in our current understanding that explain these apparent disparities, but in the absence of other explanations, I stand by our statement that based upon the evidence, the notion that the accumulation of mtDNA mutations within muscle fibers are a quantitatively meaningful contributor to muscle atrophy with aging is not compelling. Death of muscle fibers with high mtDNA mutation loads seems to be the exception rather than the rule. If further research exposes this in a manner that warrants reconsideration, I will embrace any new evidence accordingly. Notwithstanding, I am completely open to the idea that mtDNA alterations (depletion, mutation) with aging in motoneurons could contribute to the denervation observed in aging muscle and, thus, contribute to muscle atrophy indirectly. However, I am not aware of any data directly addressing this possibility and look forward to studies addressing this and other related issues. I hope this clarifies the basis for the strongly worded statement in our review [1] dismissing a quantitatively important role for mtDNA deletion accumulation within muscle fibers in causing muscle atrophy.
